# Annual Circular of the Massachusetts Medical College

**Published:** 1846-10

**Authors:** 


*Annual Circular of the Massachusetts Medical College.*—The annual circular of the Massachusetts College, Boston, embraces a history of the Medical department of Harvard College from its organization, and, also, a catalogue of those who have been graduated each year, respectively, up to the present time. It contains, in addition, lithographic prints of the new medical College, and of the Mass. General Hospital. The latter has been considerably enlarged during the last season. The new college appears to be a commodious, and finely proportioned edifice. The students at this institution have annually increased in number for several years. At the last session the class was 160.

